# Brassica yellows virus’ movement protein upregulates anthocyanin accumulation, leading to the development of purple leaf symptoms on *Arabidopsis thaliana*

**DOI:** 10.1038/s41598-018-34591-5

**Published:** 2018-11-02

**Authors:** Xiang-Ru Chen, Ying Wang, Hang-Hai Zhao, Xiao-Yan Zhang, Xian-Bing Wang, Da-Wei Li, Jia-Lin Yu, Cheng-Gui Han

**Affiliations:** 1 State Key Laboratory for Agro-biotechnology and Ministry of Agriculture Key Laboratory of Pest Monitoring and Green Management, College of Plant Protection, China Agricultural University, Beijing, 100193 P. R. China; 2 State Key Laboratory of Agro-Biotechnology and Ministry of Agriculture Key Laboratory of Soil Microbiology, College of Biological Sciences, China Agricultural University, Beijing, 100193 P. R. China

## Abstract

Poleroviruses are widely distributed and often of great economic importance because they cause a variety of symptoms, such as the rolling of young leaves, leaf color changes, and plant decline, in infected plants. However, the molecular mechanism behind these viral-induced symptoms is still unknown. Here, we verified the pathogenicity of the polerovirus Brassica yellows virus (BrYV) by transforming its full-length amplicon into *Arabidopsis thaliana*, which resulted in many abnormal phenotypes. To better understand the interactions between BrYV and its host, global transcriptome profiles of the transgenic plants were compared with that of non-transgenic Arabidopsis plants. An association between the BrYV- induced purple leaf symptoms and the activation of anthocyanin biosynthesis was noted. Using the transgenic approach, we found that movement protein of BrYV was responsible for the induction of these coloration symptoms. Collectively, our findings demonstrate the BrYV’ pathogenicity and show that the BrYV-induced purple leaf symptom resulted from its movement protein stimulating anthocyanin accumulation.

## Introduction

The family *Luteoviridae* are divided into three genera, *Luteovirus*, *Polerovirus*, and *Enamovirus*^1^. The former two genera share a similar genome organization at their 3′ ends, and their members are all phloem limited and strictly transmitted by aphids in a persistent, circulative, and non-propagative manner^2^. They are also often of great economic importance. Brassica yellows virus (BrYV) is a newly identified polerovirus^3^, which has a vast range of hosts and is widespread in mainland China, South Korea and Japan^3,4^. To date, three distinct genotypes of BrYV, BrYV-A, -B, and -C have been identified^3,5,6^.

A phylogenetic analysis showed that *Turnip yellows virus* (TuYV) is the closest relative of BrYV among the poleroviruses, with an overall nucleotide sequence identity of ~80%^3,5^. These two viruses share a similar genome organization, containing seven open reading frames (ORF0, ORF1, ORF2, ORF3a, ORF3, ORF4, and ORF5), an intergenic non-coding region (between ORF2 and ORF3a), and two untranslated regions (located at the 5′ and 3′ ends) on their positive-sense, single-strained genomic RNAs. The amino acid sequence identities of proteins encoded by the BrYVs and TuYV range from 39% to 95.5%, and the conserved P1-2, P3a, P3 (coat protein, CP) and P4 (movement protein, MP) share more than 89% identities, while P0 and P5 (read-through protein, RTP) share less than 83% and 40% identities, respectively^3,5,7,8^.

TuYV is an important virus that is extensively distributed in European countries and causes severe yield losses on oilseed rape. Oilseed rape in fields infected by TuYV often exhibits stress- and nutrient deficiency-like symptoms, such as reddening of leaf margins and interveinal yellowing and reddening^7^. Purple leaves were also observed on BrYV-infected cruciferous crops during our investigation. Owing to the close relationship between BrYV and TuYV, we hypothesized that a BrYV infection may also negatively impact crop development and production. Flavonoids are a group of ubiquitous plant secondary metabolites that have probably existed for over a billion years. They are characterized as a group of C15 scaffold molecules, formed by two aromatic cycles and a linked heterocycle, respectively, called the A-, B-, and C rings respectively. Flavonoids can be classified into several subgroups including flavonols, flavan-3-ols and anthocyanins, according C ring’s degree of oxidation^9^. In plants, these flavonoid molecules play important roles in many biological and physiological processes, such as symbiosis and flower color variation, pollinator and seed disperser recruitment, plant hormone (auxin) transport modulation, signal transduction, and biotic and abiotic (such as ultraviolet radiation, reactive oxygen species, nitrogen deficiency, and pathogen attack) stress-related defenses^10–16^.

To date, at least 54 kinds of flavonoids (35 flavonols, 11 anthocyanins and eight proanthocyanins) have been identified in Arabidopsis^17–19^. Among these flavonoid molecules, anthocyanins are well known for their anti-oxidant properties and their most visible function is the ability to turn the color of plants to red or purple^20–23^. The genetic pathway of anthocyanin biosynthesis in Arabidopsis has been well elucidated in previous works. The structural genes of anthocyanin biosynthesis are generally divided into two parts, the early biosynthesis genes such as *chalcone synthase, chalcone isomerase), flavanone 3-hydroxylase*, *and flavonoid* 3′*-hy- droxylase*, and the late biosynthesis genes, including *dihydroflavonol 4-reductase (DFR), leucoanthocyanidin oxygenase, anthocyanidin reductase*, and *UDP-glucose: flavonoid 3-O-glucosyltransferase*^24–28^. The fine regulation of anthocyanin biosynthesis is achieved by the combined reactions of several transcription factors. Early biosynthesis genes are usually regulated by a group of R2R3 MYB transcription factors, and the late biosynthesis genes are often controlled by a MYB, basic helix-loop-helix and WD40 transcription-factor complex^27,29–33^. The biosynthesis of anthocyanin can be altered by numerous biotic and abiotic stresses including light, cold, drought, sucrose, hormones, and pathogens^34–36^. Connections between these stresses and anthocyanin biosynthesis have been well studied. For example, Solfanelli *et al*. have proved that the sucrose content is closely related to the biosynthesis of anthocyanin in plants, and Lei *et al*. showed that ethylene signaling is involved in the production of anthocyanin^37,38^. The mechanistic induction anthocyanin accumulation in plants by some biotic stresses has also been revealed. For instance, Tanaka *et al*. found that *Ustilago maydis* triggered a molecular mechanism for anthocyanin induction in maize^39^.

In this work, we utilized a transgenic approach to study the pathogenicity of BrYV on one of its natural hosts, *Arabidopsis thaliana*. Transgenic Arabidopsis lines that constantly express the genomic RNA of BrYV were generated, and these transgenic lines all exhibited severe symptoms, including loss of apical dominance, shorter silique, increased numbers of rosette leaves, late flowering, and purple leaves. Subsequently, by comparing the global transcriptome profiles of transgenic lines and the control Columbia-0 (Col-0), significant transcription differenceswere found. Additionally, we tried to determine the viral cause of BrYV that resulted in the purplish color on its host leaves and found that the coloration was a consequence of BrYV’ movement protein-induced anthocyanin accumulation.

## Results

### Generation of BrYV amplicon-transformed Arabidopsis plants demonstrated the strong pathogenicity of BrYV

**Figure 1:**
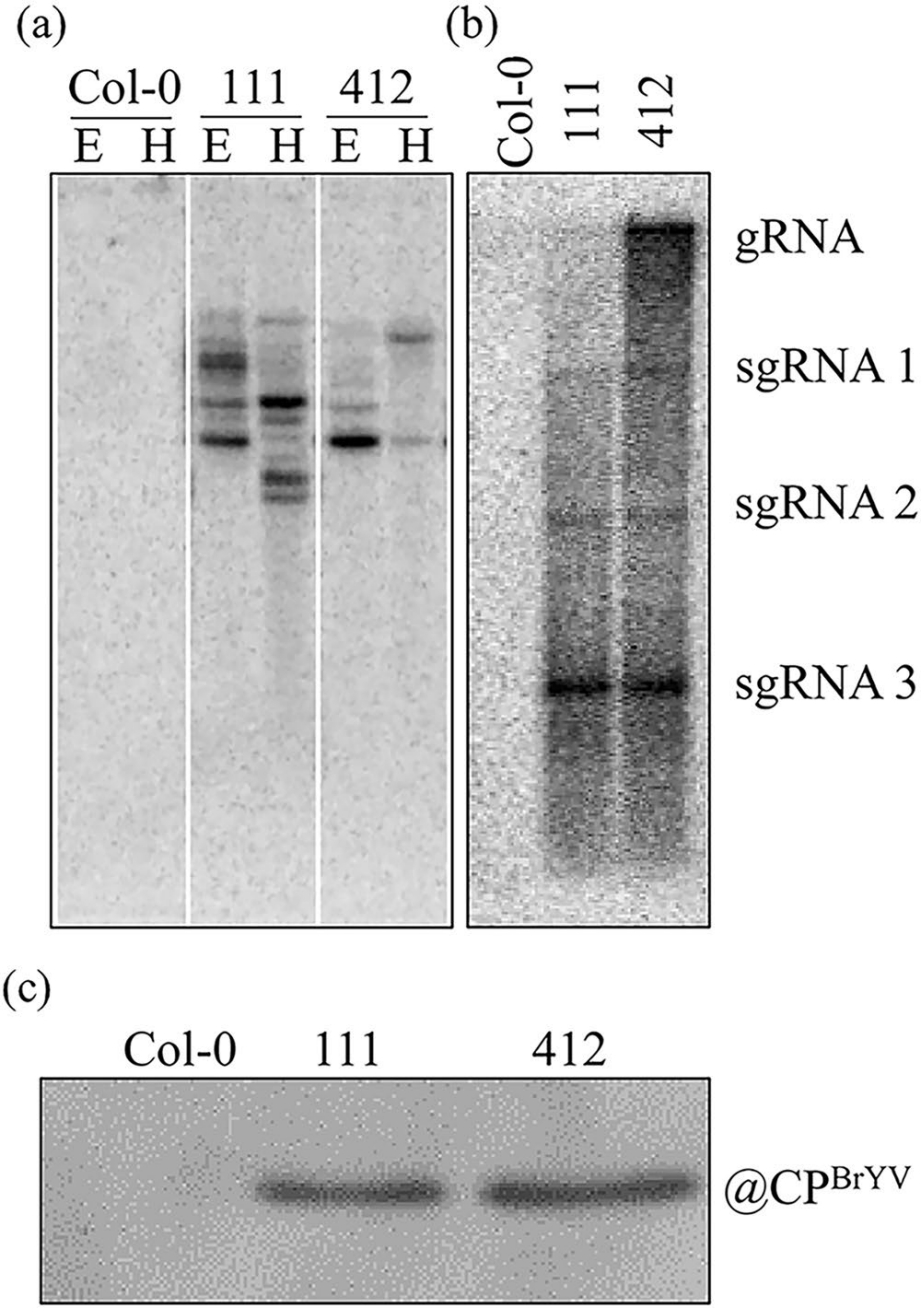
Characterization of BrYV amplicon-transformed Arabidopsis. (**a**) Southern blot analysis indicating that the cDNA of BrYV was inserted into the Arabidopsis genome. The genomic DNA of transgenic Arabidopsis plants was digested with either *Eco*R I or *Hin*d III. **(b)** Northern blot results showing the constitutive expression of BrYV-encoded genomic RNA (gRNA) and subgenomic RNAs (sgRNAs). **(c)** Western blot analysis demonstrating the expression of the BrYV coat protein in lines 111 and 412.

To better understand the interactions between BrYV and its hosts, a 35 S promoter-derived expression cassette containing the full-length cDNA of BrYV was transformed into Arabidopsis plants using the floral dip method. The plasmid pCaBrC was constructed as described in Zhang *et al*.^6^. Using hygromycin-derived rapid selection, several transgenic lines were obtained, and Southern blotting revealed that the cDNA of BrYV was successfully transformed into the Arabidopsis Col-0 ecotype (Fig. 1a). Here, we present lines 111 and 412 of these transgenic Arabidopsis plants (T4) for further study. The total RNAs of lines 111 and 412 were extracted and northern blot analyses performed (Fig. 1b). The transgenic lines were able to generate not only the genomic RNA, but also the subgenomic RNAs, of BrYV. Additionally, the CP that is translated by the subgenomic RNA of BrYV could also be detected by western blot analysis in both transgenic lines (Fig. 1c). Thus, transgenic Arabidopsis lines containing BrYV full-length amplicons were successfully developed. The BrYV amplicon was replicated, and its encoded genes were translated in these transgenic Arabidopsis lines.

**Figure 2:**
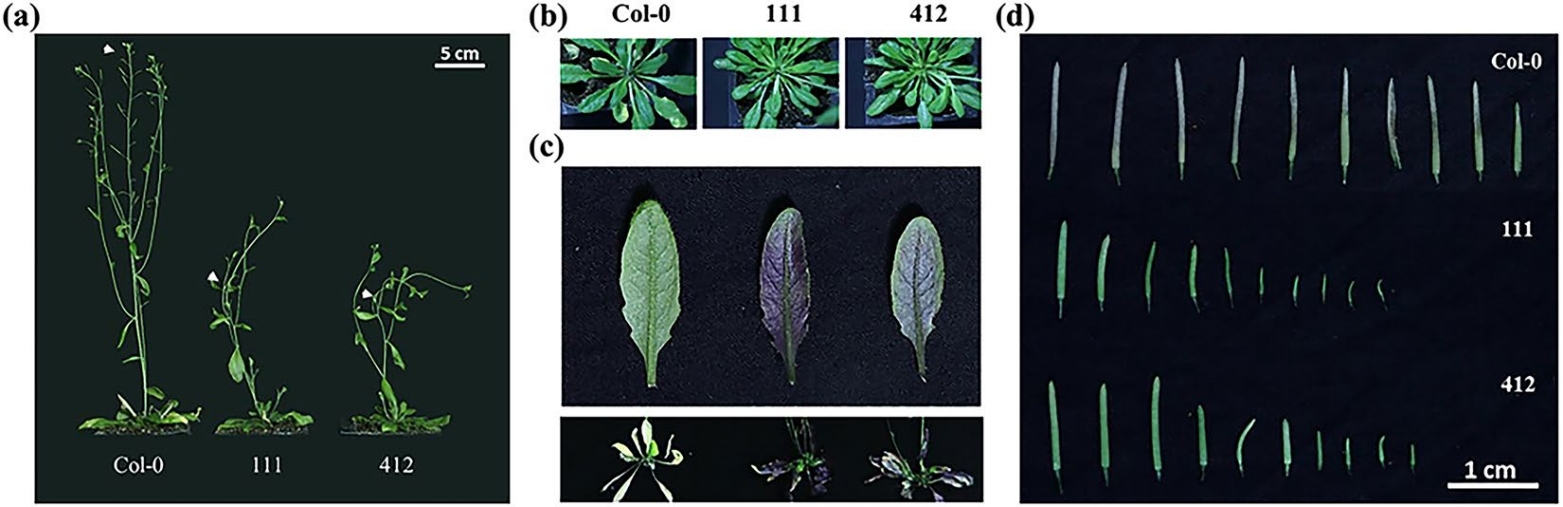
Phenotypes of BrYV amplicon-transformed Arabidopsis lines. (**a**) Dwarfism and reduced apical dominance of transgenic Arabidopsis compared with Col-0; white arrows indicate the apices of each plant. (**b**) Increased numbers of rosette leaves on transgenic Arabidopsis lines. (**c**) Purple leaf phenotype of 8-week-old (upper) and 10-week-old plants (bottom) of the BrYV amplicon-transformed Arabidopsis lines. (**d**) Silique morphology of Col-0 and the two amplicon-transformed Arabidopsis lines.

More interestingly, the expression of the BrYV amplicon in transgenic Arabidopsis lines resulted in many abnormal phenotypes compared with the Col-0 ecotype. Examples of such phenotypes included the loss of apical dominance, increased numbers of rosette leaves, late flowering, shorter silique, and the purple coloration of old leaves, which indicated that BrYV exhibited very strong pathogenicity on Arabidopsis (Fig. 2).

### Deep-sequencing-transcriptome profiles of BrYV amplicon-transformed transgenic plants

**Figure 3:**
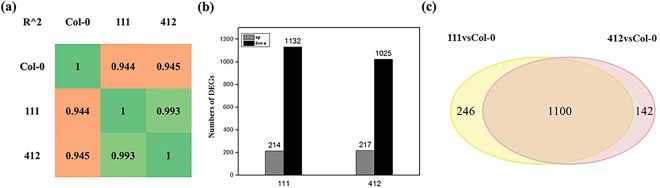
An overview of the next-generation-sequencing results of the BrYV amplicon-transformed Arabidopsis lines and Col-0. (**a**) Pearson’s correlation coefficients of the samples. (**b**) Numbers of genes up- or downregulated in lines 111 and 412. **(c)** Venn diagram showing the DEGs in the transcriptome data of lines 111 and 412.

To further characterize the molecular response of Arabidopsis to BrYV expression in transgenic lines, whole genome transcriptome profiles of wild type Col-0 and two transgenic lines, 111 and line 412, were obtained. Samples and the total RNA prepared for next-generation sequencing (NGS) are shown in the Supplemental Materials (Fig. S1). Using the HiSeq. 2000 Illumina Instrument for sequencing, more than 50 million clean reads per sample were obtained (Table 1). Pearson’s correlation analysis revealed that these samples had very similar expression patterns (Fig. 3a). Genes with greater than two fold expression changes and P values of less than 0.005 were considered as differentially expressed genes (DEGs). In total, 1,346 and 1,242 DEGs were identified from the transcriptome data of lines 111 and 412 respectively, when compared with the Col-0 wild type. More than 80% of the DEGs were upregulated in both transgenic lines (1,132 upregulated and 214 downregulated in line 111; 1,025 upregulated and 217 downregulated in line 412), indicating that the BrYV infection may activate most of the genes for the transcription of its host genome (Fig. 3b). The majority of the DEGs in lines 111 and 412 were shared and showed the same alteration trends (Fig. 3c). These mutual DEGs were most likely caused by the expression of BrYV-related genes. They were distributed in a variety of gene ontology (GO) terms, with ~55% of the GO terms being involved in biological processes, 30% in molecular function, and 15% in cellular component. The top 30 most-enriched GOs in amplicon-transformed Arabidopsis lines, and the top 20 altered DEGs, are shown in Fig. S2 and listed in Tables 2 and 3, respectivly. Other transcriptome data, including a scatterplot of the DEGs from the KEGG pathway enrichment analysis and the reads density of transcripts in chromosomes are shown in the Supplemental Materials (Figs S3 and S4). The transcriptome profiles of the BrYV amplicon-transformed transgenic plants could provide useful information on the mechanism involved in interactions between BrYV and its host.

**Table 1:** Summary of deep-sequencing results of the BrYV amplicon-transformed Arabidopsis lines and Col-0.

Index	Clean reads	Q20 (%)	GC content (%)	Total mapped	Reads map to ‘+’	Reads map to ‘−’	Non-splice reads	Splice reads
Col-0	52467312	96.7	47.01	46289246 (88.22%)	22980403 (43.8%)	22993473 (43.82%)	28980234 (55.23%)	16993642 (32.39%)
111	61938584	96.61	45.88	54647592 (88.23%)	26991146 (43.58%)	26999520 (43.59%)	32493191 (52.46%)	21497475 (34.71%)
412	57304230	96.5	45.98	50292106 (87.76%)	24959313 (43.56%)	24969592 (43.57%)	30079642 (52.49%)	19849263 (34.64%)

**Table 2:** List of top 20 significantly upregulated genes in BrYV amplicon-transformed Arabidopsis lines.

Gene ID	Gene Name	Annotation	Fold change (Up)	
			111	412
AT5G42800	DFR/TT3	Dihydroflavonol reductase	259.29	265.21
AT3G15650	—	Alpha/beta-Hydrolases superfamily protein	28.22	30.10
AT5G67100	ICU2	Putative catalytic subunit of the DNA polymerase alpha	10.18	10.13
AT1G52410	ATTSA1	Contains a novel calcium-binding repeat sequence. Binds TSK *in vitro*	10.23	9.42
AT3G60160	ABCC9/ATMRP9	Member of Multidrug Resistance Prptein subfamily	8.83	8.56
AT1G14250	—	GDA1/CD39 nucleoside phosphatase family protein	10.83	8.40
AT3G59140	ABCC10/ATMRP14	Member of Multidrug Resistance Prptein subfamily	7.79	9.66
AT4G30150	—	CONTAINS InterPro DOMAIN/s: Nucleolar 27 S pre-rRNA processing, Urb2/Npa2	7.63	8.14
AT3G28030	UVH3/UVR1	Required for repair of pyrimidine-pyrimidinone (6–4) dimers	7.57	7.45
AT1G14460	—	AAA-type ATPase family protein	8.18	7.34
AT3G54670	ATSMC1/TTN8	Encodes a member of the Arabidopsis cohesin complex that is essential for viability and sister chromatid alignment.	7.42	7.07
AT4G30990	—	ARM repeat superfamily protein	7.91	6.97
AT4G15210	BAM5/RAM1	Cytosolic beta-amylase expressed in rosette leaves and inducible by sugar	7.38	6.94
AT3G11964	RRP5	Encodes a nucleolar protein that is a ribosome biogenesis co-factor	6.87	7.04
AT2G42270	—	U5 small nuclear ribonucleoprotein helicase, putative	6.77	7.47
AT5G61190	—	Putative endonuclease or glycosyl hydrolase	6.67	6.68
AT1G06720	—	P-loop containing nucleoside triphosphate hydrolases superfamily protein	6.73	6.64
AT1G22060	—	FBD, F-box and Leucine Rich Repeat domains containing protein	6.68	6.51
AT1G62310	—	Transcription factor jumonji (jmjC) domain-containing protein	6.94	6.49
AT1G77580	—	Plant protein of unknown function (DUF869)	6.44	6.81

**Table 3:** List of top 20 significantly downregulated genes in BrYV amplicon-transformed Arabidopsis lines.

Gene ID	Gene Name	Annotation	Fold change (Down)	
			111	412
AT4G34410	ERF109/RRTF1	Encodes a member of the ERF (ethylene response factor) subfamily B-3 of ERF/AP2 transcription factor family	−17.74	−208.51
AT1G74930	ORA47	Encodes a member of the DREB subfamily A-5 of ERF/AP2 transcription factor family	−14.67	−30.64
AT3G48360	ATBT2	Encodes a protein (BT2) that is an essential component of the TAC1-mediated telomerase activation pathway	−8.05	−8.12
AT5G52050	ATDTX50	MATE efflux family protein	−7.66	−9.86
AT3G15450	—	Aluminium induced protein with YGL and LRDR motifs	−6.40	−9.82
AT4G24570	DIC2	Encodes one of the mitochondrial dicarboxylate carriers	−5.85	−9.57
AT2G40610	ATEXP8	Member of Alpha-Expansin Gene Family. Naming convention from the Expansin Working Group	−5.52	−5.28
AT4G27450	—	Aluminium induced protein with YGL and LRDR motifs	−5.03	−8.19
AT1G64360	—	—	−5.45	−4.93
AT1G74670	GASA6	Gibberellin-regulated family protein	−4.85	−5.50
AT2G25735	—	—	−4.66	−9.45
AT1G69890	—	Actin cross-linking protein	−4.45	−5.14
AT2G25900	ATCTH/ATTZF1	Encodes a protein with two tandem-arrayed CCCH-type zinc fingers that binds RNA and is involved in RNA turnover	−4.44	−5.40
AT3G04640	—	Glycine-rich protein	−4.38	−7.34
AT4G29780	—	—	−4.34	−7.31
AT5G20630	ATGER3/GLP3	Encodes a germin-like protein. Its transcripts are more abundant in RNA from leaves collected in the evening	−6.14	−4.33
AT2G27385	—	Pollen Ole e 1 allergen and extensin family protein	−4.25	−5.47
AT2G33830	ATDRM2	Dormancy/auxin associated family protein; CONTAINS InterPro DOMAIN/s	−4.18	−4.98
AT3G15630	—	—	−4.18	−4.79
AT1G80440	KFB20/KMD1	The KISS ME DEADLY (KMD) family protein.	−3.94	−6.20

### Expression of the BrYV amplicon increased the anthocyanin content and induced the upregulation of genes involved in anthocyanin biosynthesis in leaves of BrYV amplicon-transformed plants

**Figure 4:**
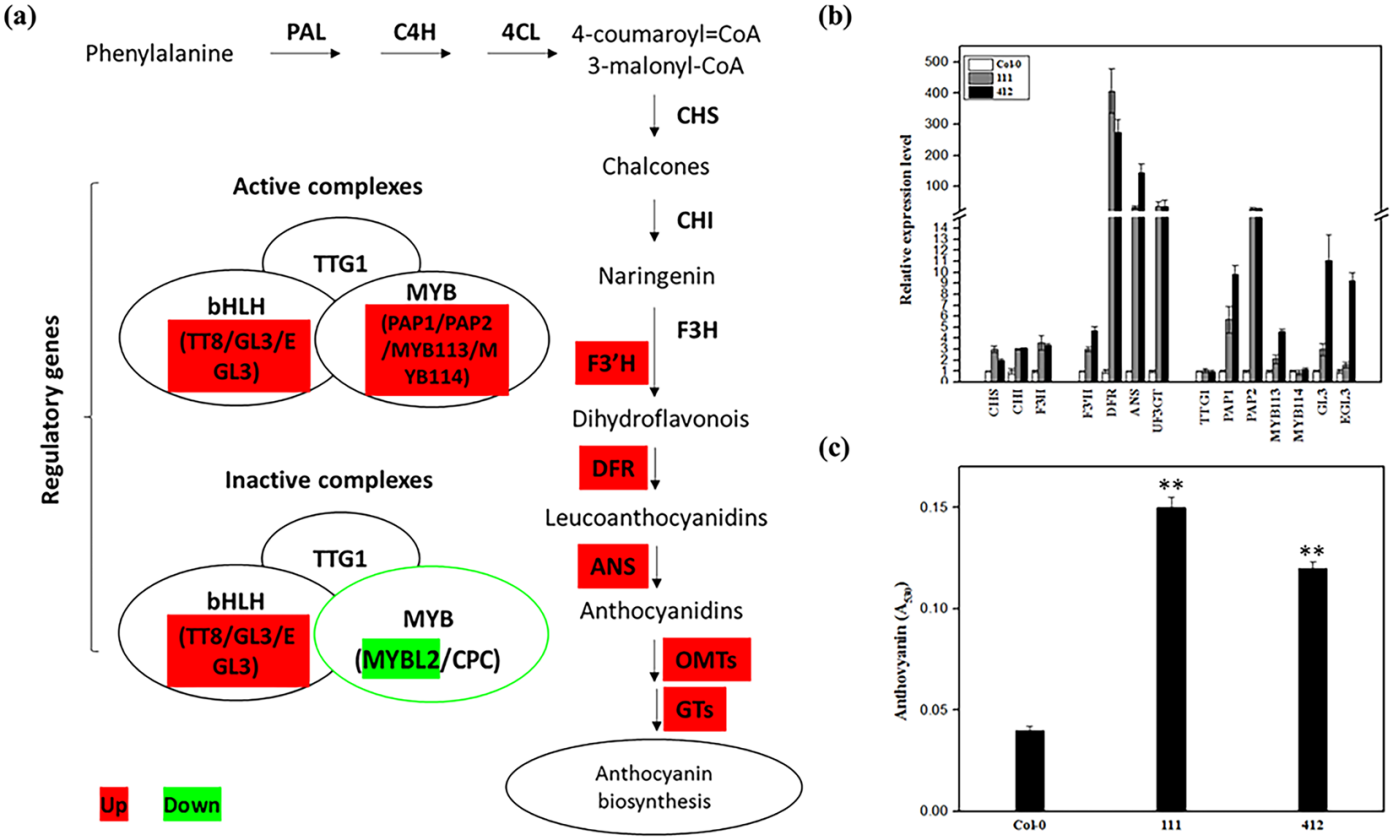
Anthocyanin biosynthesis analysis of Col-0, and lines 111 and 412. (**a**) Schematic diagram of the anthocyanin biosynthetic pathway modified based on Viola *et al*.^41^. Genes up- and downregulated in the pathway are shaded in red and green, respectively. (**b**) Transcript levels of structural genes early biosynthesis genes, chalcone synthase, *CHS*, chalcone isomerase, *CHI*, flavanone 3-hydroxylase and flavonoid 3′-hydroxylase, *F3H*, and late biosynthesis genes dihydroflavonol 4-reductase, *F*3′*H*, leucoanthocyanidin oxygenase, *DFR*, anthocyanidin reductase, *ANS*, and UDP-glucose:flavonoid 3-O-glucosyltransferase, *UF3GT*, and regulatory genes (*TTG1*, *PAP1*, *PAP2*, *MYB113*, *MYB114*, *GL3*, and *EGL3*) involved in anthocyanin biosynthesis detected by quantitative RT-qPCR using total RNA extracted from 6-week-old Col-0, line 111 and line 412. The p-value of each gene did in this test was listed in the Table S2. **(c)** Anthocyanin contents in 8-week-old seedlings of Col-0, 111 and 412. Mean ± SD of n = 3 independent experiments. **P < 0.01.

The *DFR* gene, which is involved in the flavonoid biosynthetic pathway, was dramatically upregulated in both BrYV amplicon-transformed Arabidopsis lines, compared with the wild eco-type Col-0. By the late stage of their life cycle, the color of the leaves of these BrYV amplicon-transformed Arabidopsis lines turned purple (Fig. 2c). The color changes of plant leaves were caused by the co-expression of many pigments, and anthocyanin are usually responsible for the formation of purple^40^. The anthocyanin biosynthetic pathway in Arabidopsis has been well-elucidated^41^. We first analyzed the NGS data to gain an overview of the expression levels of the genes involved in the anthocyanin biosynthetic pathway (Fig. 4a and Table S1). Then to validate the results of the transcriptome data and to verify whether the anthocyanin biosynthetic pathway was actually upregulated in BrYV amplicon-transformed Arabidopsis, we examined the expression level of genes involved in the anthocyanin biosynthetic pathway using RT-qPCR analysis. As expected, the majority of the genes involved in the anthocyanin biosynthetic pathway were upregulated (Fig. 4b and Table S2). We then detected the anthocyanin content in lines 111 and 412 using the method described in Joeng *et al*.^42^. The anthocyanin contents in lines 111 and 412 were indeed greater than in the wild type Col-0 (Fig. 4c).

### The expression of sucrose biosynthesis-related genes were upregulated with anthocyanin accumulation in BrYV amplicon-transformed transgenic lines

**Figure 5:**
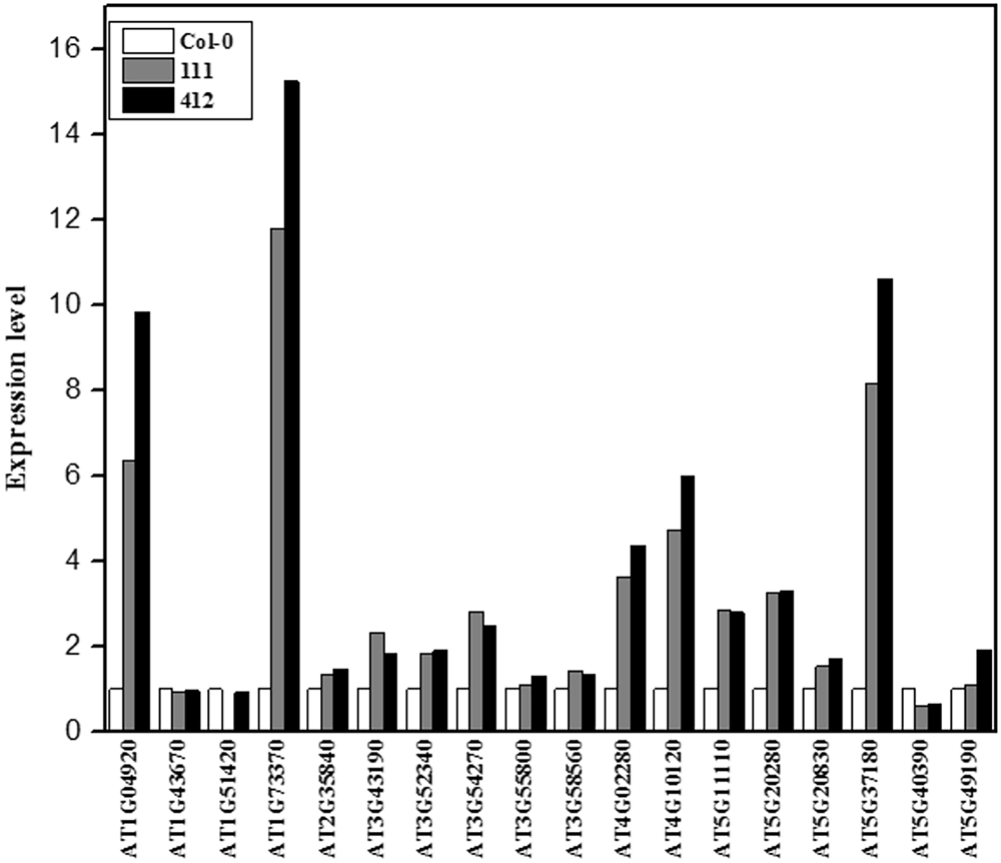
Alterations in the sucrose biosynthetic pathways in Col-0 and two BrYV amplicon-transformed Arabidopsis lines. Genes involved in sucrose biosynthesis were identified based on their annotated Gene Ontology categories; http://amigo.geneontology.org/amigo/term/GO:0005986. The expression data per gene was generated by transcriptome data.

The biosynthesis of anthocyanins are closely related to the sucrose contents of plants^37,43–45^. To determine whether the induction of anthocyanin biosynthesis in BrYV amplicon-transformed Arabidopsis was activated by sucrose, we checked the expression levels of genes involved in the sucrose biosynthetic pathway (Fig. 5 and Table S3). Most of the genes involved in the biosynthetic pathway of sucrose were upregulated, indicating that the increase of anthocyanin content in BrYV amplicon-transformed Arabidopsis was related to sucrose stress.

### Generation of transgenic Arabidopsis lines containing the *MP* gene alone further indicated that *MP* alone could trigger the development of the purple leaf symptom by upregulating the anthocyanin biosynthetic pathway

**Figure 6:**
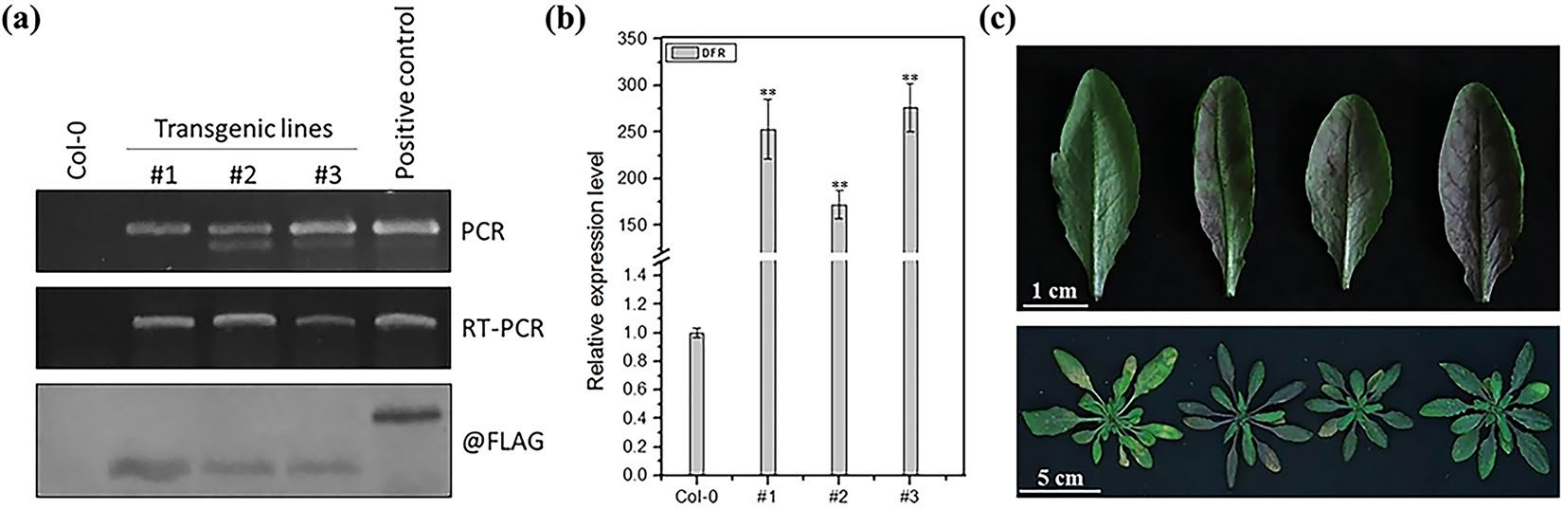
Ectopic expression of MP^BrYV^ changes the phenotype of transgenic Arabidopsis lines. (**a**) Molecular analysis of transgenic lines (#1, #2, and #3) that consistently expressing the MP^BrYV^. For PCR detection the positive control was the pCaBrC plasmid. For RT-PCR detection, a sample of BrYV-infected *Nicotiana benthamiana* was used for positive control. For the western blot analysis, the positive control was a sample transiently expressing the FLAG-tagged P0 protein. (**b)** RT-qPCR analysis of *DFR* expression levels in #1, #2, and #3. Mean ± SD of n = 3 independent experiments. **P < 0.01. **(c)** Morphological characteristics of MP^BrYV^-transformed Arabidopsis lines. Upper: 8-week-old leaves of Col-0, #1, #2, and #3; bottom: 8-week-old plants of Col-0 and the three MP^BrYV^-transformed Arabidopsis lines.

**Figure 7:**
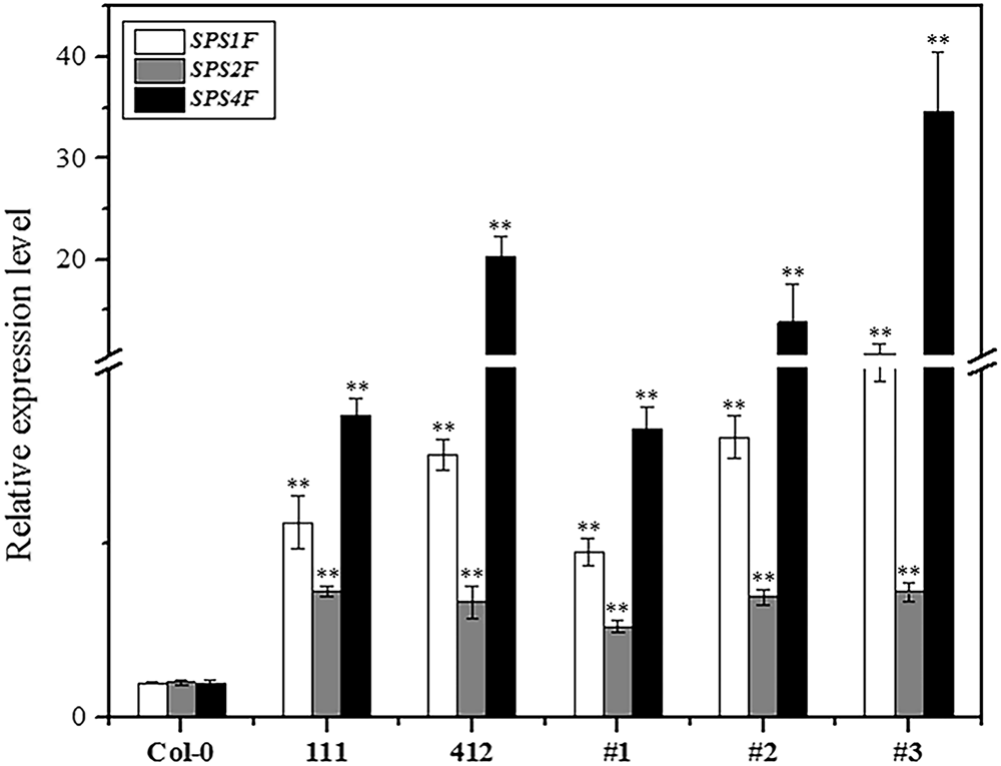
RT-qPCR analysis of sucrose-phosphate synthases expression in BrYV amplicon and MP^BrYV^-transformed Arabidopsis lines. SPS1F, SPS2F, and SPS4F are three sucrose-phosphate synthases essential for sucrose biosynthesis in Arabidopsis and they were all upregulated in both amplicon- and MP^BrYV^-transformed transgenic Arabidopsis lines. Mean ± SD of n = 3 independent experiments. **P < 0.01.

Because TuYV, the virus related to BrYV, could also induce the purple leaf symptom on oilseed rape, we hypothesized that their highly similar 3′ genome sequences may be of great importance for anthocyanin accumulation in plants infected by one of these viruses. Furthermore, the biosynthesis rates of sucrose in *Potato leaf roll virus*-encoded MP-transformed potato and tobacco plants were upregulated^46,47^. Because sucrose can act as a kind of stress factor that effects the accumulation of anthocyanin in plants, we believed that the MP of BrYV could also share this ability, which could lead to the upregulation of anthocyanin in BrYV-infected cruciferous plants. To test our hypothesis, transgenic Arabidopsis plants constantly expressing the MP^BrYV^ protein were generated by *Agro-bacterium-*mediated floral dip transformations with the vector pMDC32-MP-3FLAG. Using rapid hygromycin selection, RT-PCR detection, and western blot analyses, three independent transgenic lines, #1, #2, and #3, that constantly expressed the Flag-tagged MP^BrYV^ protein were obtained (Fig. 6a). We evaluated the expression levels of the *DFR*, which is a crucial structural gene involved in the anthocyanin biosynthetic pathway that was dramatically upregulated in the amplicon-transformed Arabidopsis plants, in lines #1, #2, and #3 (Fig. 6b). As shown in Fig. 6b, the expression levels of *DFR* were obviously upregulated. Furthermore, the MP^BrYV^-transformed Arabidopsis lines also exhibited a purple leaf phenotype (Fig. 6c). We also detected the expression levels of three sucrose-phosphate synthase genes (*SPS1F/AT5G20280*, *SPS2F/AT5G11110*, and *SPS4F/AT4G10120*), which are crucial for sucrose biosynthesis in Arabidopsis plants and they were all up-regulated in the NGS data. The RT-qPCR showed that all three genes were upregulated in both BrYV amplicon- and MP^BrYV^-transformed Arabidopsis lines (Fig. 7), suggesting that the upregulation of anthocyanin biosynthesis might be stimulated by MP through a sucrose-dependent pathway in BrYV-infected plants.

### Phytohormones may not participate in the stimulation of anthocyanin production in BrYV-infected plants

**Figure 8:**
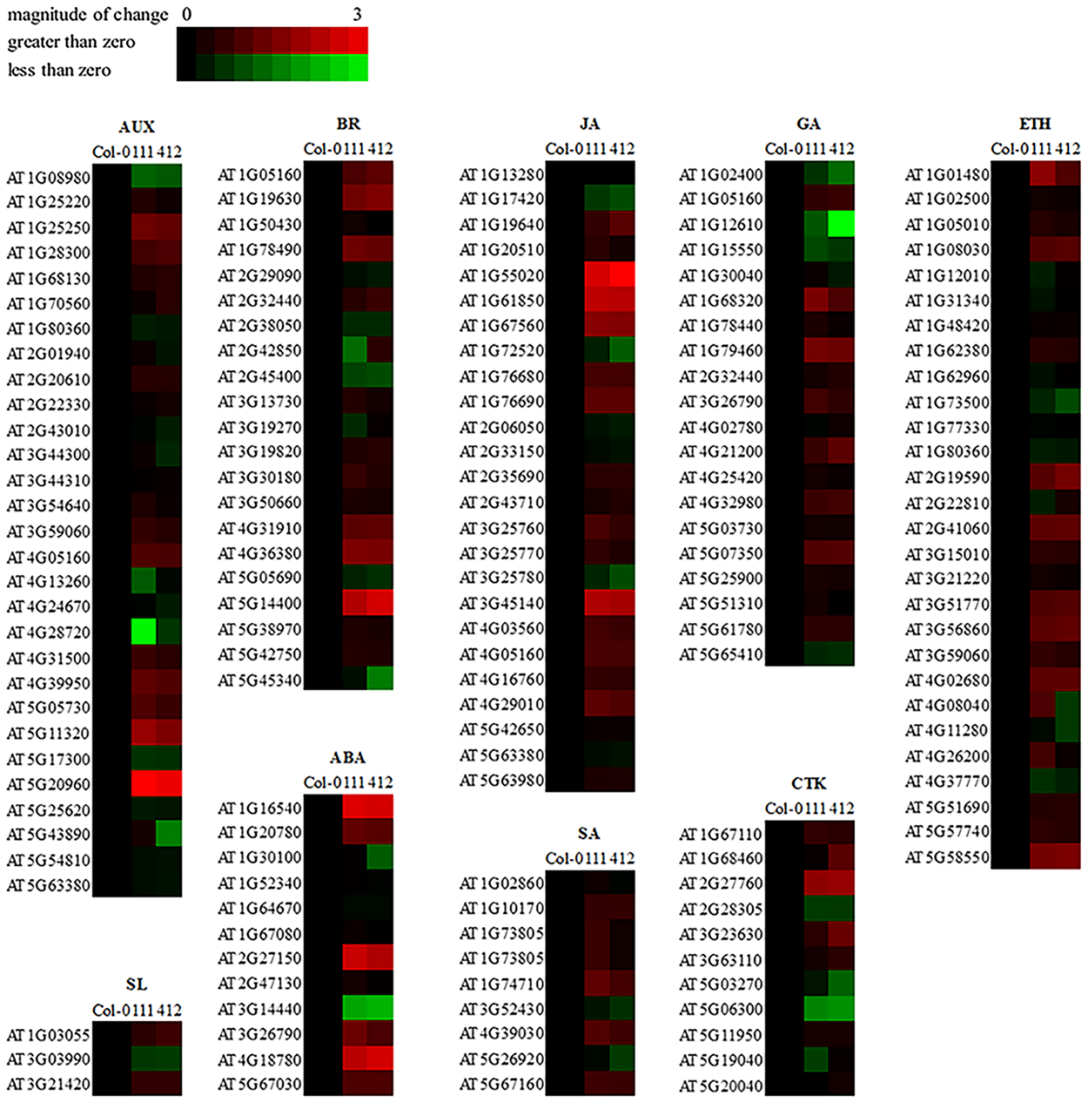
Expression profiles of hormone biosynthesis-related genes in BrYV amplicon-transformed Arabidopsis lines. Genes involved in the biosynthesis of plant hormones, including abscisic acid (ABA), auxin (AUX), brassinosteroid (BR), cytokinin (CTK), ethylene (ETH), gibberellin (GA), jasmonate (JA), salicylic acid (SA), and strigolactone (SL), were identified based on their annotated Gene Ontology categories (AUX: GO 0009851, SL: GO 1901601, BR: GO 0016132, ABA: GO 0009688, JA: GO 0009695, SA: GO 0009697, GA: GO 0009686, CTK: GO 0009691, ETH: GO 0009693). The heatmap was constructed using the BAR HeatMapper Plus Tool based on the log_2_ fold change of each gene involved in plant hormone biosynthesis, with a maximal value of 3.

Plant hormones have a great impact on the biosynthesis of anthocyanins^44,45^. To determine whether the accumulation of anthocyanin in BrYV amplicon-transformed Arabidopsis plants was associated with specific plant hormones, we analyzed the expression levels of the genes involved in the biosynthesis of nine major phytohormones, abscisic acid (ABA), auxin (AUX), brassinosteroid (BR), cytokinin (CTK), ethylene (ETH), gibberellin (GA), jasmonate (JA), salicylic acid (SA), and strigolactone (SL). The transcriptome data showed that the biosynthetic pathways of these nine main phytohormones were changed in the amplicon-transformed Arabidopsis lines compared with Col-0, implying that the BrYV infection could disturb the well-organized phytohormone network in its hosts, and this disturbance might be directly linked to many of the abnormal phenotypes exhibited on these transgenic lines (Fig. 8 and Table S4).

Subsequently, we selected nine genes that were involved in phytohormone biosynthetic pathways and examined their expression levels in Col-0, line 111, line 412 and MP^BrYV^-transformed Arabidopsis. The RT-qPCR results showed that all nine selected genes in the amplicon-transformed lines showed the same alteration trends as they did in the transcriptome data (Table 4). This result further confirmed the reliability of the NGS data, which indicated that the biosynthesis of plant hormones in these transgenic plants was modulated by BrYV and that changes in the biosynthesis of phytohormones might result in the exhibition of the abnormal phenotypes on the transgenic plants. Additionally, the expression levels of most of these genes in the MP^BrYV^-transformed Arabidopsis lines were not obviously changed compared with in the Col-0 ecotype, suggesting that the phytohormones may not participate in the stimulation of anthocyanin production in BrYV-infected plants.

**Table 4:** Fold changes of genes involved in phytohormone biosynthesis in MP- and amplicon-transformed Arabidopsis plants compared with Col-0.

Phytohormone	Gene ID	Gene name	Transcriptome	qRT-PCR	
			Amplicon (Fold change)	Amplicon (Fold change)	MP (Fold change)
ABA	AT1G16540	*AtLOS5*	5.74 ± 0.20	3.94 ± 0.25	2.19 ± 0.26
AUX	AT5G11320	*AtYUCCA4*	3.07 ± 0.32	1.84 ± 0.38	0.62 ± 0.07
BR	AT4G36380	*AtROT3*	2.72 ± 0.04	4.43 ± 0.25	1.61 ± 0.34
CTK	AT2G27760	*AtIPT2*	3.27 ± 0.13	4.43 ± 0.49	1.25 ± 0.23
ETH	AT4G02680	*AtEOL1*	2.09 ± 0.02	1.57 ± 0.44	1.01 ± 0.11
GA	AT1G79460	*AtGA2*	4.41 ± 0.21	3.38 ± 0.24	1.04 ± 0.17
JA	AT1G55020	*AtLOX1*	7.61 ± 1.36	1.63 ± 0.33	0.79 ± 0.08
SA	AT4G39030	*AtEDS5*	1.79 ± 0.15	2.26 ± 0.37	1.27 ± 0.16
SL	AT3G21420	*AtLBO1*	1.49 ± 0.01	1.44 ± 0.15	0.96 ± 0.20

Data are the averages of three biological replicates per sample.

## Discussion

In this study, we demonstrated the strong pathogenicity of the BrYV polerovirus by transforming its full-length amplicon into Arabidopsis (Col-0 ecotype). Many abnormal phenotypes, including dwarfism, loss of apical dominance, shorter silique, and purplish leaves were found on the amplicon-transformed Arabidopsis lines (Fig. 2). To reveal how BrYV could induce these abnormal phenotypes on its hosts, global transcriptome profiles of two BrYV amplicon-transformed transgenic Arabidopsis lines (111 and 412) and the wild type Col-0 were obtained. NGS results revealed that 1,488 genes involved in a broad range of physiological, biochemical, and metabolic processes were differentially expressed in the BrYV amplicon-transformed Arabidopsis lines. The majority of them shared the same variation tendency, and they were distributed in a variety of pathways including hormone, flavonoid, and sucrose biosynthesis (Figs 3 and S3), indicating that these genes may participate in the interactions between BrYV and its hosts. Among these shared DEGs, the vast majority were upregulated in transgenic lines when compared with Col-0 (Fig. 3b). This was mainly the result of the transcription and translation of BrYV, which had elicited the expression of some genes that were shut down or poorly expressed under natural conditions.

The normal growth and development of plants are dependent on the appropriate biosynthesis and accurate co-regulation of different phytohormones. However, plant pathogens often manipulate the plant hormone biosynthetic pathways or interfere with hormone signals to facilitate their own infection processes^48^. When the balance of this highly sophisticated hormone regulatory network is disturbed, abnormal phenotypes usually appear. For example, the loss of apical dominance on *Turnip vein clearing virus* infected Arabidopsis is related to the content of AUX in plants^49^; the dwarf symptom induced by *Rice dwarf virus* on rice plants is a result of P2 *Rice dwarf virus* protein-triggered decrease in gibberellin biosynthesis^50^. The interactions between plant hormones and viruses are well documented in a previous review^51^. The NGS data in our study showed that, the expression patterns of many genes involved in the biosynthesis of the nine main phytohormones in BrYV amplicon-transformed Arabidopsis were altered (Fig. 8). Thus, the abnormal phenotypes exhibited on these Arabidopsis plants might also be evoked by the perturbation of phytohormone biosynthesis or their signal transduction. For example, AUX is the leading factor controlling the apical dominance of plants^52,53^. The significantly downregulated gene *AtERF109* listed in Table 3 was reported to mediate the cross-talk between JA and AUX in Arabidopsis^54^. *AtEDS5*, which is involved in the biosynthesis of salicylic acid was upregulated in both the transcriptome data and RT-qPCR analysis (Table 4). The over-expression of *AtEDS5* not only enhances resistance to viruses but also leads to dwarf symptoms on Arabidopsis plants^55^. *AtROT3*, which we analyzed in this study (Table 4), may participate in the manipulation of leaf shape^56^. The genes mentioned above and the plant hormones to which they are connected, might be involved in the abnormal phenotypes exhibited on the BrYV amplicon-transformed Arabidopsis plants, but further work still needs to performed to uncover the correlations between the abnormal phenotypes and BrYV-modulated phytohormones. In addition, some P0 proteins encoded by poleroviruses have been characterized as strong RNA-silencing suppressors, which can cause developmental defects in plants by disrupting miRNA functions. Thus, the abnormal phenotypes on the BrYV-transgenic plants may result from the disruption of miRNA functions by a BrYV-encoded P0 suppressor^57^.

The purple coloration of host tissues caused by plant pathogens has been widely reported. For example, anthocyanin streaking can be observed on *Ustilago zeae*- and *Sorosporium reilianum*-inoculated maize variety B164^58,59^; “Candidatus Phytoplasma asteris” OY-W strain (OY-W phytoplasma) can induce purple discoloration on Arabidopsis and Petunia plants^60^; the geminivirus *Beet curly top virus* can cause purple coloration on virus-infected plant tissues^61^; and reddish-purple leaves have been found on *Grapevine leafroll-associated virus-3*-infected grapevines^62,63^. Many viruses belonging to *Luteoviridae* can also induce purple coloration symptoms on their host plants. For instance, TuYV, which is widespread in European countries, induces reddening/purpling of the leaves on oilseed rape^64^; reddish tinge can be found on some *Sugarcane yellow leaf virus*-infected sugarcane cultivars^65^; and the red leaf disease on oats and maize is caused by a luteovirus called *Barley yellows dwarf virus*^66,67^. Here, we showed that the transformation of the BrYV amplicon can induce purple leaf symptoms on transgenic Arabidopsis plants. Recently, the connection between plant pathogens and their associated purple coloration of the host has been elucidated. Gutha *et al*. found that, the *Grapevine leafroll-associated virus-3* infection could upregulate many genes involved in anthocyanin biosynthesis and particularly a transcription factor named MybA1, which could induce the accumulation of anthocyanin in plants by positively regulating the expression of a structural gene involved in anthocyanin biosynthesis named *UFGT*^68^; Sanchez-Lopez *et al*. verified that *Alternaria alternata* emits volatile compounds, a group of metabolites with low polarity, high vapor pressure, and molecular masses less than 300 Da, could trigger anthocyanin accumulation by accelerating the biosynthesis of cytokinins in its hosts^69^. Furthermore, Tanaka *et al*. demonstrated that Tin2 effector of *U. maydis* can induce the anthocyanin biosynthesis in maize by masking the ubiquitin-proteasome degradation motif in the kinase ZmTTK1, resulting in its stabilization. ZmTTK1, controls the activation of genes involved in anthocyanin biosynthesis^39^. In this paper, we revealed that the MP of BrYV is the viral determinant responsible for the acceleration of anthocyanin accumulation in BrYV amplicon-transformed Arabidopsis plants.

The significant effects of anthocyanin accumulation on the infective processes of plant pathogens in their hosts has also been previously investigated. Serrano *et al*. showed that flavonoids and their derivatives have repressive roles in microbe-associated molecular pattern-triggered host immunity^70^. The increased anthocyanin biosynthesis triggered by Tin2, depletes the precursor of lignin biosynthesis, leading to a decreased lignification of vascular bundle cells, and finally results in a maize plant that is much more vulnerable to *U. maydis* infection^39^. We surveyed the transcriptome data and found no obvious alterations in the gene expression states in the lignin biosynthetic pathway, indicating that, anthocyanin accumulation in BrYV-infected plants may operate in a way unlike that in *U. maydis*-infected maize. Himeno *et al*. demonstrated that the activation of anthocyanin biosynthesis in Arabidopsis can suppress the cell death caused by OY-W phytoplasma infection^60^. Coincidently the MP of a plant virus within the same family as BrYV, named *Barley yellow dwarf virus*, may trigger the programed cell death (PCD) on tobacco leaves in a dose-dependent manner^71^. Thus, the anthocyanin accumulated in BrYV-infected Arabidopsis may function as an anti-oxidant like it does in OY-W phytoplasma-infected Arabidopsis, whereas no sign of PCD was found on the transgenic Arabidopsis plants generated in this study, implying that there might be an integrative relationship among the anthocyanin concentration, the viral titter and PCD in plants. We highly suspect that the accumulation of anthocyanin in transgenic plants is a result of the expression of MP^BrYV^, because previous studies confirmed that MP *Potato leaf roll virus* expression could alter the carbohydrate levels in transgenic plants by increasing the plasmodesmal permeability in mesophyll cells^46,47,72^. Aadditionally, the sucrose content is closely related to the biosynthesis of anthocyanin in plants^37^. Link *et al*., showed that the phosphorylation of S71/S79 is essential for the plasmodesmata localization of MP ^*Potato leaf roll virus*^^73^; however, the association between the phosphorylation and its function in inducing the accumulation of anthocyanin has not yet been confirmed. Furthermore, host proteins that directly interact with the MP protein should be identified to determine how the MP protein induces anthocyanin accumulation.

Here, we demonstrated the pathogenicity of BrYV because the amplicon-containing transformants exhibited severe abnormal phenotypes and greatly changed transcriptome profiles. A NGS analysis of these BrYV amplicon-transformed Arabidopsis provided abundant genetic resources for studying the interactions between poleroviruses and their hosts. We further discovered that MP^BrYV^ was the viral determinant of the purple coloration symptom on BrYV-infected plants and that it acted by upregulating anthocyanin biosynthesis. However the exact molecular MP^BrYV^-modulated regulatory mechanism of anthocyanin biosynthesis during the BrYV infection of its hosts needs to be further investigated.

## Materials and Methods

### Plasmid construction

The plasmid containing a full-length cDNA amplicon of BrYV-C, called pCaBrC, was constructed by Zhang *et al*.^6^, based on pCass4-Rz^74^. pCaBrC was used to generate transgenic lines that constitutively expressed the full-length viral genomic RNA of BrYV. The nucleotide sequence encoding the MP of BrYV was amplified directly from pCaBrC using specific primers and inserted into the binary vector pMDC32-3FLAG using *Apa* I and *Spe* I restriction sites^75^. This plasmid was named pMDC32-MP-3FLAG. All of the plasmids were verified by DNA sequencing (Tsingke Biotech, Beijing) and then introduced into *Agrobacterium tumefaciens* strain C58CI. The primers used in plasmid construction are listed in the Table S5.

### Plant materials and growth conditions

The Col-0 ecotype of *A. thaliana* was utilized to generate stable transgenic Arabidopsis plants. Six-week-old Arabidopsis plants grown in a greenhouse at 22 °C, with 16 h light/8 h dark cycle, 100–120 μmol m^−2^ s^−1^ light intensity, and 65% relative humidity were used for RNA-seq, RT-qPCR, blot analyses, and anthocyanin content evaluation.

### Plant transformation

Two plasmids, pCaBrC and pMDC32-MP-3FLAG were chosen to generate transgenic Arabidopsis plants using the *Agrobacterium*-mediated floral dip transformation method as described previously^76^, and the rapid selection of positive T1 offspring was carried out as per Samuel^77^.

### Nucleic acid extraction

The total RNA of Col-0 wild type and transgenic lines were extracted from the rosette leaves of 6-week-old Arabidopsis plants, using TRIzoL reagent, as per the manufacturer’s manual (Invitrogen). For each sample, at least eight individual Arabidopsis plants were pooled together to form a biological replicate. For RT-qPCR and northern blot analyses, the quality of RNA was detected by 1% agarose gels (Biowest) and Nanodrop 2000 (Thermo). For RNA-seq the concentration and integrity of the total RNA was further assessed by Beijing Novogene Bioinformatics Technology Co. Ltd. (BNBTC), Beijing, China, using the RNA Nano 6000 Assay Kit of the Bioanalyzer 2100 system (Agilent Technologies).

Genomic DNA of Arabidopsis wild type (Col-0) and transgenic lines were isolated from the rosette leaves of 6-week-old Arabidopsis seedlings using a CTAB procedure^78^.

### RNA-seq

Total RNA of three samples, Col-0 and two amplicon-transformed Arabidopsis T4 lines (111 and 412), were isolated. For each sample, the total RNA of three biological replicates were mixed and sent to BNBTC for NGS to produce a transcriptome profile. BNBTC prepared the cDNA library and sequenced the samples. In total, 3 μg RNA per sample was used as input. Sequencing libraries were generated using the NEBNextR Ultra^TM^ RNA library Prep Kit for Illumina (NEB, USA), 150–200 bp cDNA fragments were preferentially purified with the AMPure XP system (Beckman Coulter, Beverly, USA), amplified by PCR, clustered using a TruSeq PE Cluster Kit v3-cBot-HS (Illumina) and finally sequenced on an Illumina HiSeq platform.

### Bioinformatics analysis

For quality control, raw data in fastq format were first processed through in-house perl scripts. Here, the raw reads containing adapters and poly-Ns, as well as low-quality reads, were removed to produce clean data. Additionally, the Q20, Q30 and GC content were calculated. All of the downstream analyses were based on the high-quality clean data.

GO and KEGG analyses of DEGs, an index of the *A. thaliana* (Col-0) genome was built using Bowtie v2.2.3, and paired-end clean reads were aligned to the genome using TopHat v2.0.12. HTSeq v0.6.1 was used to count the read numbers mapped to each gene. The FPKM value of each gene was calculated based on the length of the gene and the read counts mapped to this gene^79^. For the DEGs analysis, the read counts of each gene were first adjusted by the edgeR program package using one scaling normalized factor and then analyzed by a DEGSeq R package (1.20.0) with a P-value of less than 0.005^80^. The GO enrichment analysis of DEGs was implemented by the GOseq R package, with a corrected P value of less than 0.05. KOBAS software was used to determine the statistical enrichment of DEGs in KEGG pathways.

### RT-qPCR

To validate the results of the RNA-seq, sets of specific primers for the gene expression analysis were synthesized. Some were obtained from the previous paper, while others were newly designed^42^.

RT-qPCR was performed as previously described with slight modifications^81,82^. The total RNA, extracted as described above, was treated with DNase I (TaKaRa, Dalian, China) for 1 h at 37 °C to eliminate genomic DNA contamination. Approximately 3 μg total RNA per sample was used for cDNA synthesis using SMART^®^ M-MLV Reverse Transcriptase (Promega, Madison, USA). The amplification program was as follows: 5 min at 95 °C, followed by 38 cycles of 5 s at 95 °C and 30 s at 60 °C, and after the cycles, a final step at 72 °C for 30 s was applied. The endogenous gene which encodes the ACTIN2 protein of *A. thaliana* was the reference gene in this study, and the values of the target genes were normalized to *ACTIN2*. Primers used for RT-qPCR are listed in Table S5. The RT-qPCR tests were conducted three independent times.

### Southern blot analysis

The protocol established by E. M. Southern for Southern blot analysis was used to confirm the transformation events. Total genomic DNA (5 μg) of Arabidopsis seedlings was digested with *Eco*R I-HF or *Hin*d III-HF (New England Biolabs) overnight at 37 °C, fragments were separated by gel electrophoresis, transferred to N+ membrane (Amersham Biosciences, Roosendaal, The Netherlands) using the capillary transfer method, hybridized with a radioactive isotopes [α-32P] dCTP-labeled cDNA probe specific for nt 5,161 to 5,620 of the 3′ BrYV fragment, recorded by phosphor autoradiography, and finally scanned using a Typhoon 9000 (GE Healthcare). Primers used to amplify the probes are listed in Table S5.

### Northern blot analysis

For the detection of BrYV RNAs generated by the BrYV amplicon-transformed Arabidopsis lines, 2 μg total RNA of Col-0, line 111, and line 412 were prepared and fractionated by electrophoresis with ~5 V/cm force in a denaturing agarose gel containing formaldehyde. The northern blot analysis was performed with a [α-32P] dCTP-labeled cDNA probe specific for nt 5,161 to 5,620 of the 3′ BrC fragment as described previously^83,84^.

### Western blot analysis

Western blotting was performed following the protocols of Wang *et al*. and Zhuo *et al*. did^85,86^. In total, 0.1 g leaves per sample were ground into powder in liquid nitrogen and mixed with 400 μL 2× SDS buffer [100 mM Tris (pH 6.8), 20% glycerol, 4% SDS, and 0.2% bromophenol blue] containing 10% β-mercaptoethanol. The samples were then boiled at 100 °C for 5 min, and centrifuged at 12,000 × g for 5 min before being loaded on a gel. Proteins were separated by 12.5% SDS-PAGE and then transfered to Hybond-C membranes. The membranes were blocked overnight in TBST buffer (150 mM NaCl, 10 mM Tris–HCl, pH 8.0, and 0.05% Tween-20) plus 5% nonfat dried milk and then incubated for 4 h at room temperature with specific polyclonal antibodies. The membranes were washed with TBST buffer three times and incubated with a 1:5,000 diluted Protein A-alkaline phosphatase (Sigma-Aldrich) in TBST. Finally, the target proteins (CP^BrYV^ in lines 111 and 412, and FLAG-tagged MP in #1, #2, and #3) were detected with BCIP/NBT substrates (Sigma-Aldrich).

### Evaluation of anthocyanin

The measurement of anthocyanin content was done as described previously^42,87^, and this test was verified for three times. Anthocyanin was extracted by shaking 0.1 g leaves per sample overnight at 4 °C in 600 μL acidic methanol (1% HCl in methanol, v/v). After extraction, 400 μL of water and 400 μL of chloroform were added to the extract and mixed. After centrifugation at 12,000 × g for 2 min, the absorbance levels of the supernatant was measured at 530 and 657 nm, and the concentration of anthocyanin were calculated using A_530_−0.25 A_657_.

## Electronic supplementary material

Supplementary figures

Supplementary tables
