# Supplementary material for: Brassica yellows virus’ movement protein upregulates anthocyanin accumulation, leading to the development of purple leaf symptoms on Arabidopsis thaliana
Source: Sci Rep. 2018 Nov 2;8:16273. doi: 10.1038/s41598-018-34591-5 (PMC6215002; doi:10.1038/s41598-018-34591-5)
Supplement: Supplementary file 1 — Supplementary figures [file 41598_2018_34591_MOESM1_ESM.docx]

**Brassica yellows virus’ movement protein upregulates anthocyanin accumulation, leading to the development of purple leaf symptoms on *Arabidopsis thaliana***

Xiang-Ru Chen^1^, Ying Wang^1^, Hang-Hai Zhao^1^, Xiao-Yan Zhang^1^, Xian-Bing Wang^2^, Da-Wei Li^2^, Jia-Lin Yu^2^, Cheng-Gui Han^1^*

^1^State Key Laboratory for Agro-biotechnology and Ministry of Agriculture Key Laboratory of Pest Monitoring and Green Management, College of Plant Protection, China Agricultural University, Beijing 100193, P. R. China

^2^State Key Laboratory of Agro-Biotechnology and Ministry of Agriculture Key Laboratory of Soil Microbiology, College of Biological Sciences, China Agricultural University, Beijing 100193, P. R., China

*Correspondence to Cheng-Gui Han Email address：hanchenggui@cau.edu.cn


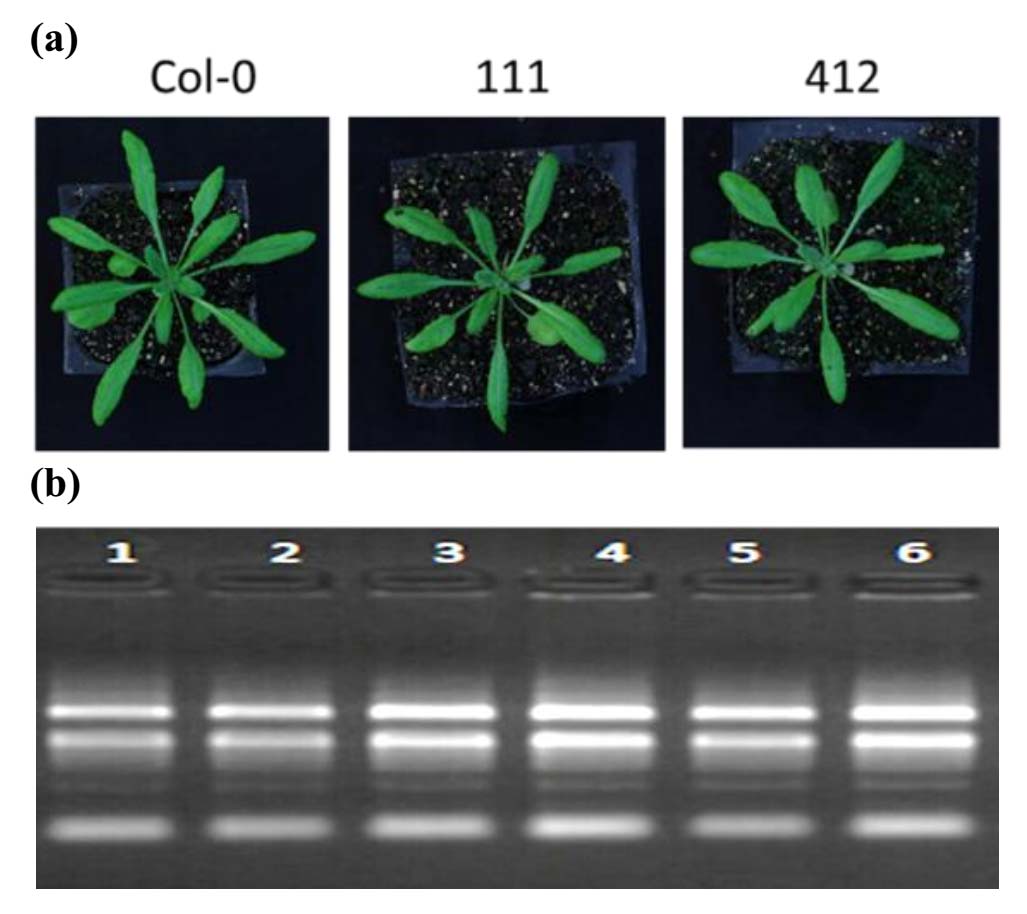


**Figure S1. Samples prepared for next generation sequencing.**


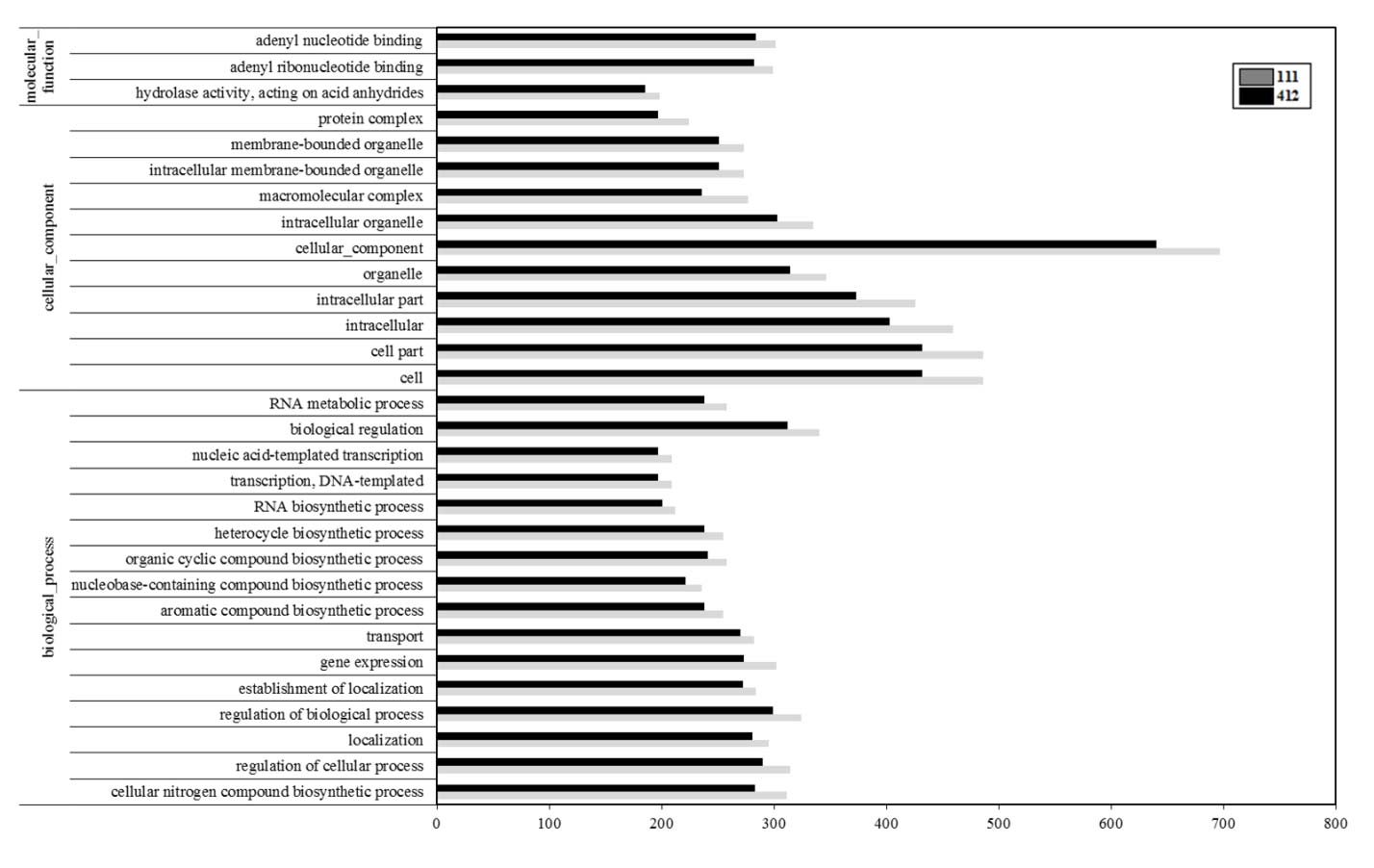


**Figure S2. Top 30 GO terms enriched in the BrYV amplicon-transformed Arabidopsis lines (111 and 412) compared with Col-0.**


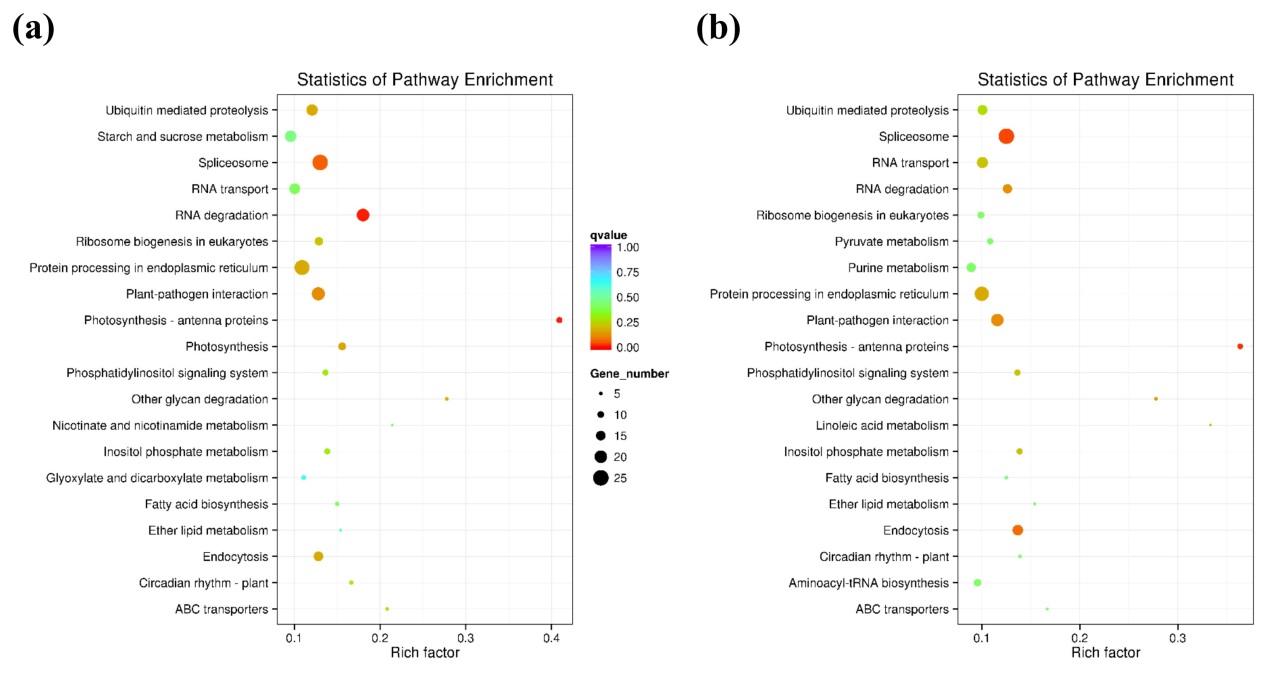


**Figure S3. Scatter plot of top 20 enriched KEGG pathways in lines 111 and 412.**


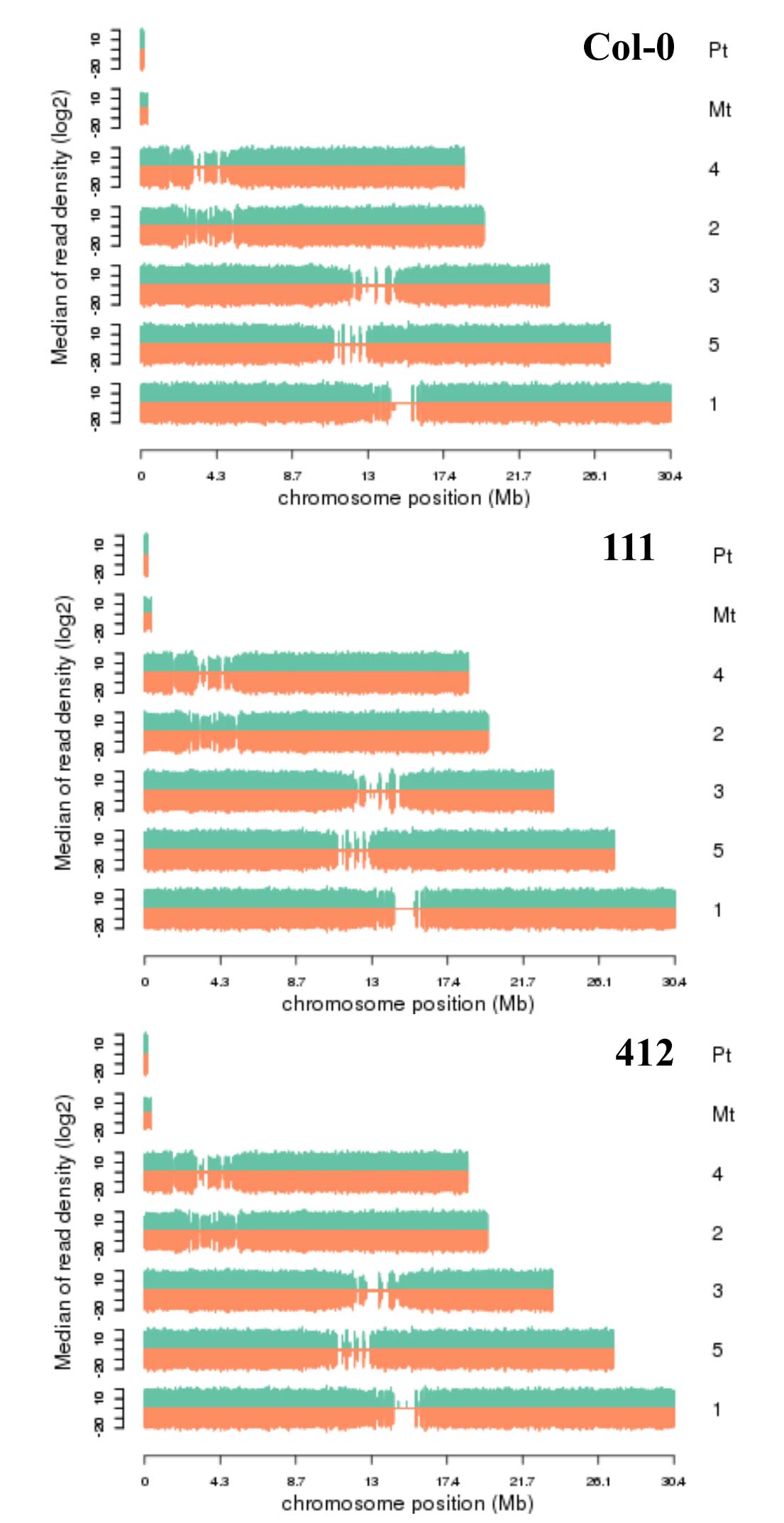


**Figure S4. Reads density of transcripts on chromosomes.**
